# Genu Recurvatum Deformity in a Child due to Salter Harris Type V Fracture of the Proximal Tibial Physis Treated with High Tibial Dome Osteotomy

**DOI:** 10.1155/2012/219231

**Published:** 2012-08-13

**Authors:** Theodoros Beslikas, Andreas Christodoulou, Anastasios Chytas, Ioannis Gigis, John Christoforidis

**Affiliations:** 2nd Orthopaedic Department, General Hospital “G.Gennimatas”, Aristotle University of Thessaloniki, 54635 Thessaloniki, Greece

## Abstract

Salter-Harris type V fracture is a very rare injury in the immature skeleton. In most cases, it remains undiagnosed and untreated. We report a case of genu recurvatum deformity in a 15-year-old boy caused by a Salter-Harris type V fracture of the proximal tibial physis. The initial X-ray did not reveal fracture. One year after injury, genu recurvatum deformity was detected associated with significant restriction of knee flexion and limp length discrepancy (2 cm) as well as medial and posterior instability of the joint. Further imaging studies revealed anterior bone bridge of the proximal tibial physis. The deformity was treated with a high tibial dome osteotomy combined with a tibial tubercle osteotomy stabilized with malleolar screws and a cast. Two years after surgery, the patient gained functional knee mobility without clinical instability. Firstly, this case highlights the importance of early identification of this rare lesion (Salter-Harris type V fracture) and, secondly, provides an alternative method of treatment for genu recurvatum deformity.

## 1. Introduction

Fractures of the proximal tibia are among the rarest of physeal injuries, accounting for between 0.5 and 3% of all injuries involving the growth plate [1–3]. Many authors suggest that the proximal growth plate of the tibia is less prone to injury because of the lack of any significant attachment of the collateral ligaments. Furthermore, the epiphysis is buttressed laterally by the head of the fibula and anteriorly by the forward projection of the tibial tuberosity [1, 4–6]. Salter-Harris type V fractures of proximal tibial physis are even rarer. These lesions can lead to genu recurvatum deformity if misdiagnosed and mistreated. Osseous genu recurvatum is most often due to asymmetrical growth arrest of the proximal tibial physis affecting primarily the tibial tubercle [7]. Restoration of the deformity can be achieved either by gradual correction with the Ilizarov technique or by acute correction after bony osteotomies [7]. Several osteotomies have been described in the literature with open and closed wedge osteotomies being the most prominent. The purpose of our study is to present a case of genu recurvatum caused by a Salter-Harris type V fracture of the proximal tibial physis treated with a high tibial dome osteotomy in combination with tibial tubercle osteotomy.

## 2. Case Report

**Figure 1:**
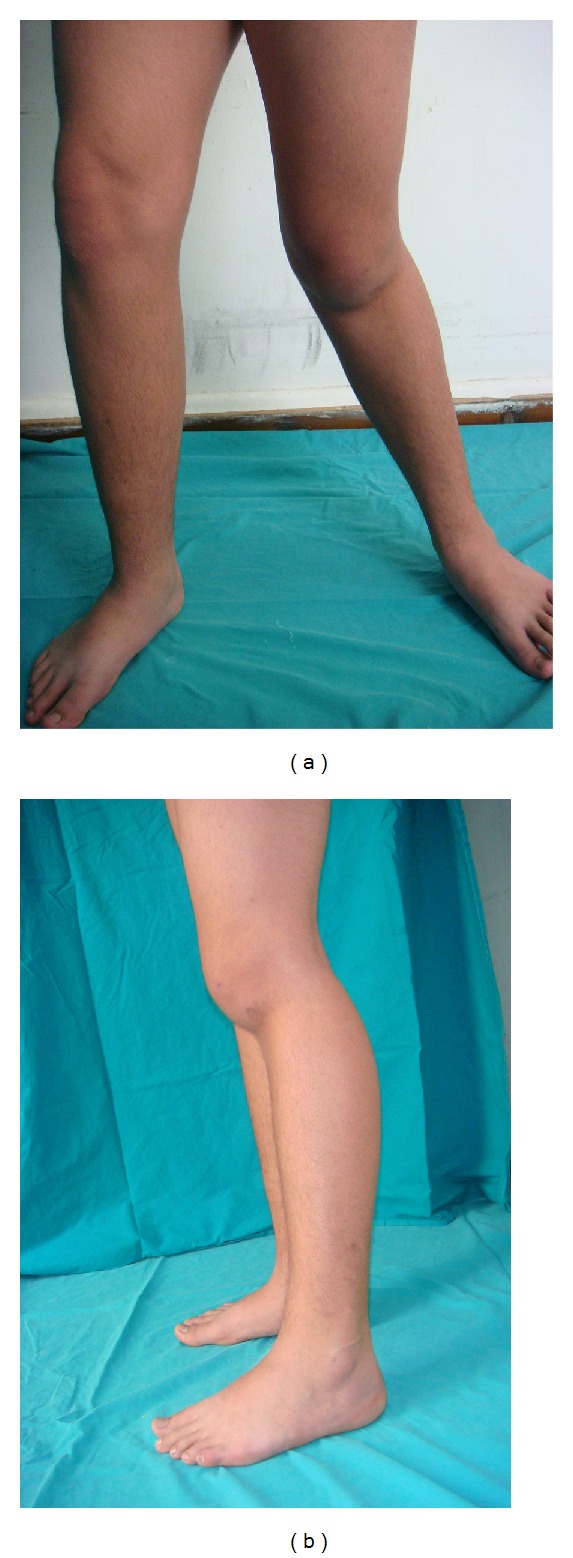
Clinical appearance of the left knee one year after the injury: medial instability and posterior subluxation of the left knee—genu recurvatum.

**Figure 2:**
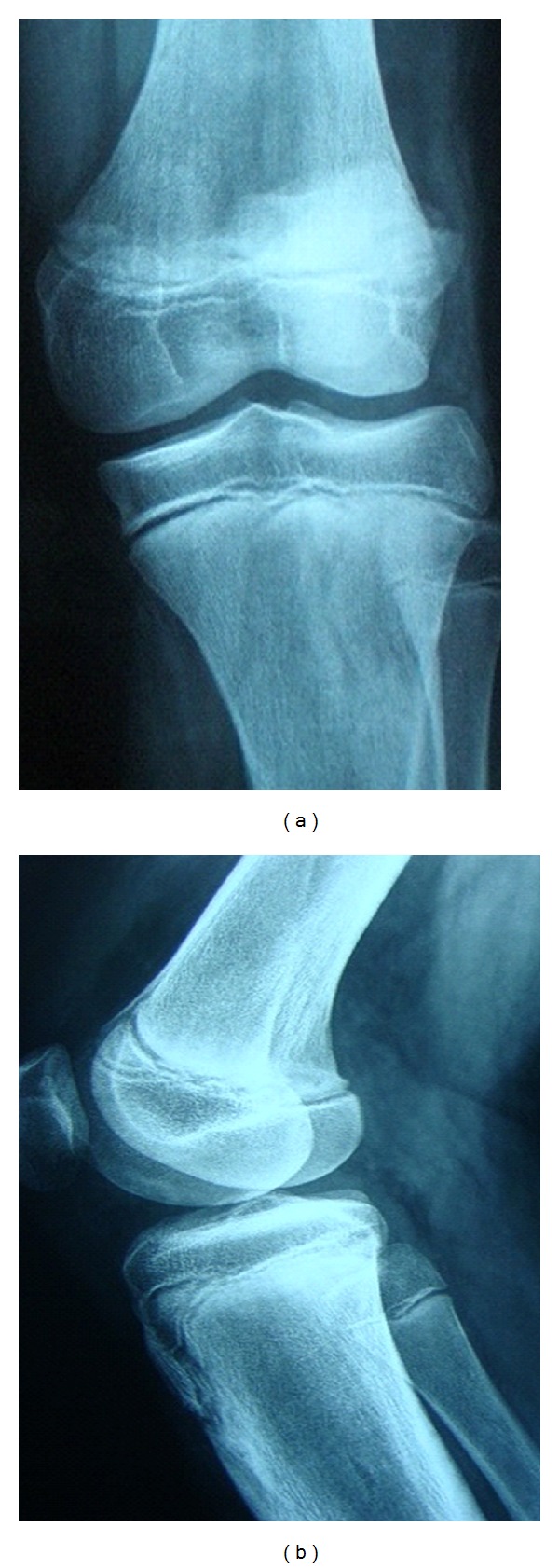
Initial X-rays of the injured knee: no evidence of fracture—separation.

A 15-year-old boy with a history of left knee injury presented in our outpatient clinic limping using armpit crutches. Clinical examination revealed genu recurvatum deformity, posterior and medial joint instability, and limb length discrepancy (2 cm). Knee flexion was limited significantly (less than 25°) (Figure 1). The initial injury that was associated with haemarthrosis was treated a year ago with elastic bandage stabilization for 2 weeks in another hospital. Early mobilization was recommended after the completion of the treatment. The primary X-ray control demonstrated no bone injury (Figure 2).

**Figure 3:**
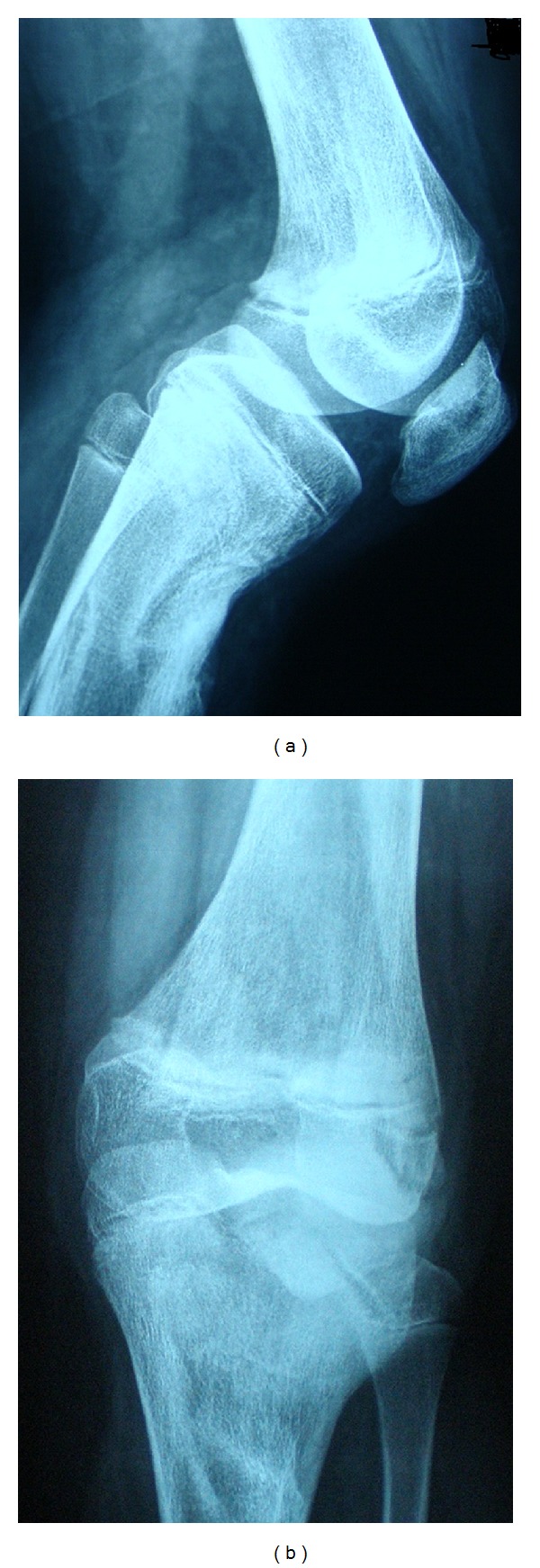
X-rays of the left knee one year after the injury: proximal tibial growth plate arrest associated with tibial tubercle growth plate arrest. Posterior subluxation and recurve left knee joint. Genu recurvatum deformity 30°.

**Figure 4:**
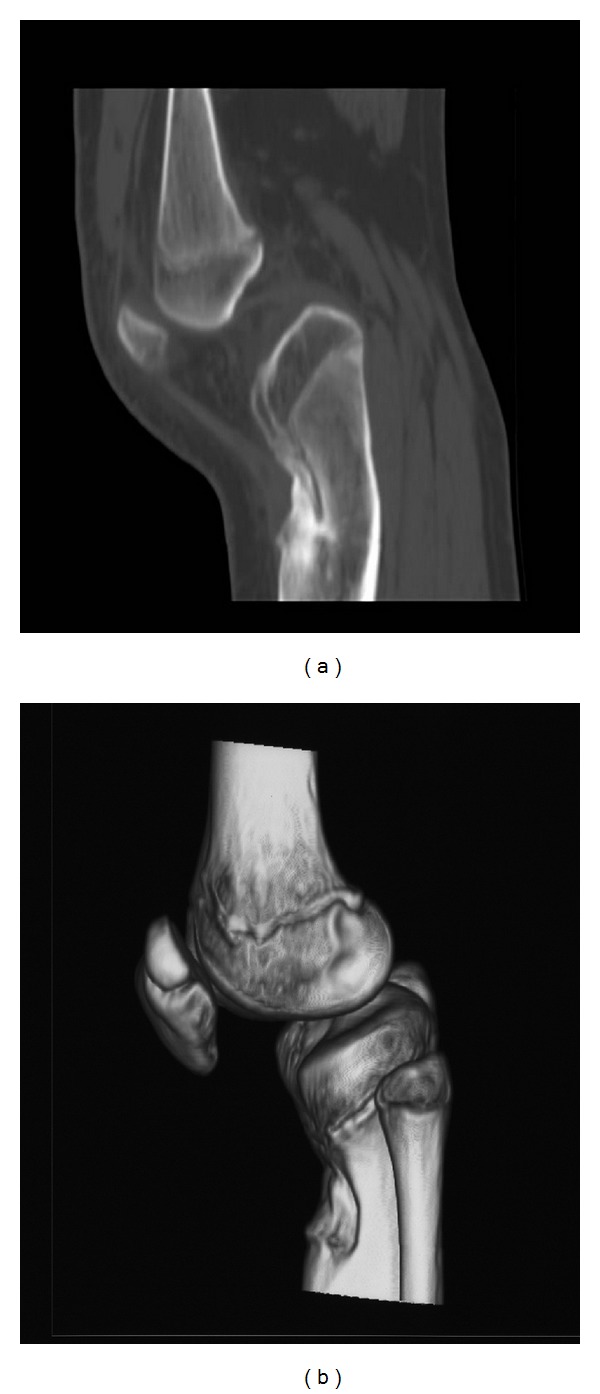
CT tomography of the left knee: posterior subluxation of the knee and significant deformity of the tibial tubercle.

**Figure 5:**
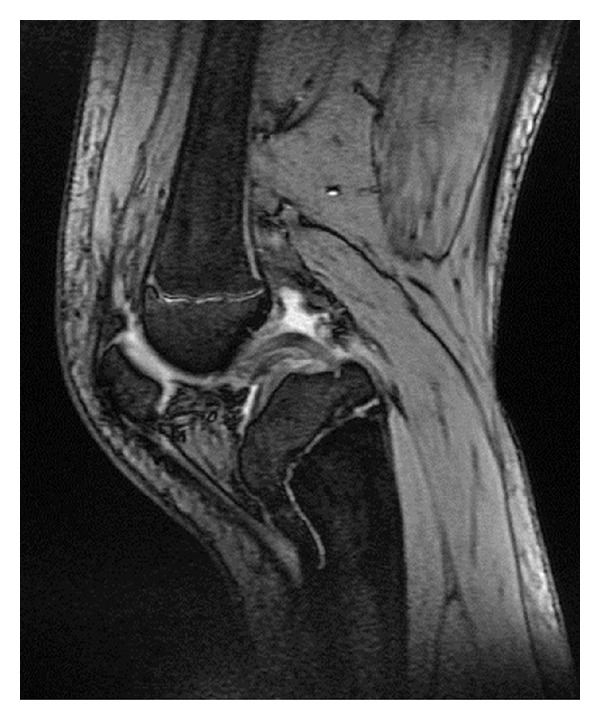
MRI of the left knee: narrowing of the proximal tibial growth plate, arrest of the tibial tubercle, thickening and shortening of the extensor mechanism, relaxation of the posterior cruciate ligament, and posterior subluxation of the joint.

**Figure 6:**
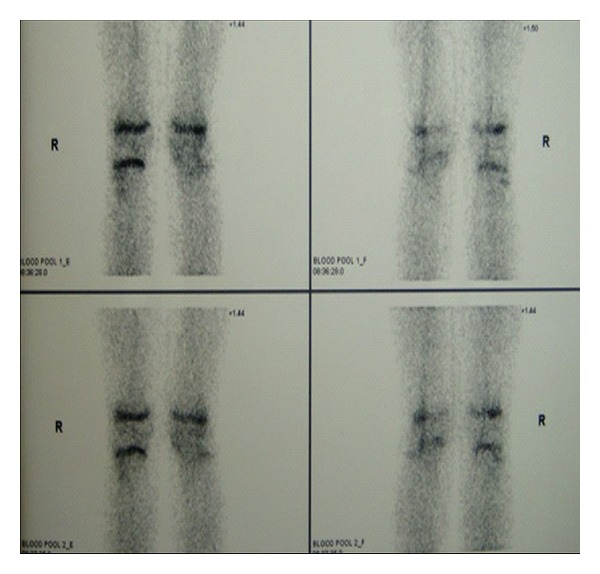
Bone scanning with ^99m^Tc-MDP: reduced perfusion of the proximal tibial growth plate of the left knee.

Our X-ray control demonstrated subluxation of the joint, osseous recurvatum deformity of 30° affecting the proximal left tibia, fuzziness in the anterior proximal part of the growth plate and a tibial tubercle hypoplasia (Figure 3). CT and three-dimensional CT of the left knee revealed bone bridge over the anterior proximal tibial epiphysis—proximal tibial metaphysis and severe recurvatum deformity. The tibial tubercle was displaced posteriorly accompanied by angulation of the tibial plateau (Figure 4). MRI of the affected knee demonstrates physeal arrest of the tibial tubercle. The proximal tibial growth plate was undisturbed while the patellar tendon thickened (Figure 5). ^99m^Tc-MDP bone scan revealed no evidence of tumor or inflammation of the left knee. The growth plate of the proximal tibia presented with reduced perfusion and osteoblastic function (Figure 6). 

High tibial dome osteotomy, proximal to the tibial tubercle, was performed. Tibial tubercle osteotomy followed. Iliac bone autograft was used to restore the tibial tubercle and elongate the extensor mechanism. The tibial and the tubercle osteotomies were stabilized with two malleolar screws with washers and a long leg cast. The cast was replaced after 6 weeks with a functional knee brace that permitted active knee exercises. Weight bearing was allowed 3 months after operation when union of the osteotomy was achieved. 

**Figure 7:**
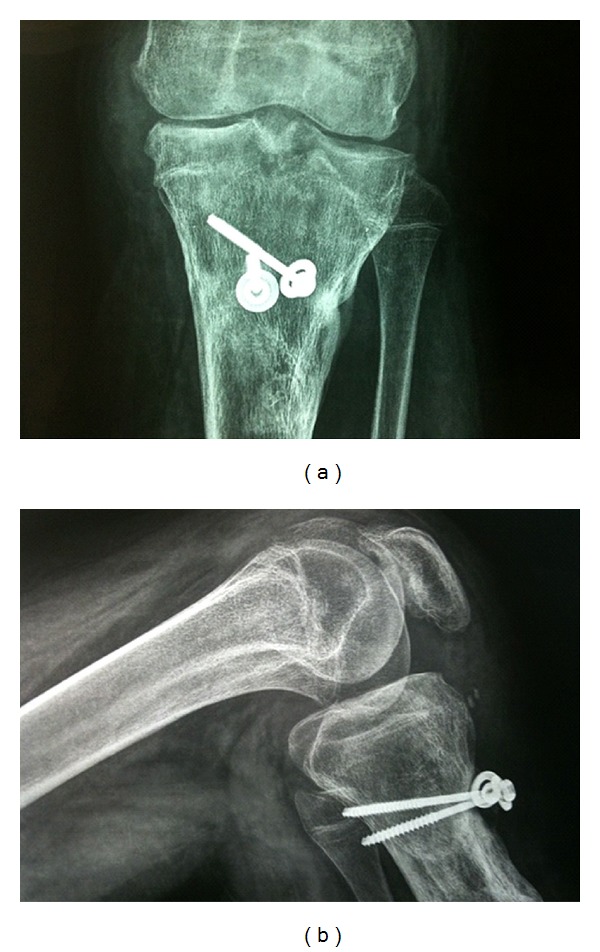
X-rays of the left knee two years after the procedure.

Two years after the procedure, the patient had full knee extension, flexion of 120°, and restoration of the normal axis (Figure 7). Preoperative posterior and medial instability were restored without any ligamentous reconstruction. 

## 3. Discussion

Salter-Harris type V fractures were described by Salter and Harris as a crush injury to the physis with initially normal X-rays with late identification of premature physeal closure. Epiphyseal plate injuries occur during childhood mainly because of local trauma. However, physeal plate injuries about the knee do not necessarily appear initially, even with nonphyseal fractures of the lower extremities [8]. According to Ogden [9], closure of the proximal tibial physis is complete by 13–15 years in girls and 15–19 years in boys. The physis is particularly vulnerable to injury just before this time. The region under the tuberosity is the last portion to close. The Salter-Harris type V physeal injury is difficult, if not impossible, to diagnose acutely. The only radiographic indication may be a decrease in the normal width of the radiolucent physis. Radiographs of the affected and the contralateral knee should be compared especially with regard to the thickness and configuration of the physis. These fractures are usually diagnosed in retrospect, long after the injury [10]. The development of a bony bar, within some months following the initial injury, is associated with complete arrest of growth of only a portion or of the entire physis. This eventually produces a joint surface deformity, an angular deformity, and/or a length discrepancy of the affected extremity. MRI seems to have an important role in the early evaluation and staging of acute pediatric growth plate injuries, as well as in the assessment of growth arrest [11–15]. The early identification of a traumatic physeal injury may lead to easier management because the treatment aims at resolving the physeal arrest rather than addressing both the arrest and an acquired growth deformity [10].

Genu recurvatum deformity is a rare condition which may be caused by bone or soft tissue pathology (capsuloligamentous recurvatum) in the area of the knee or both [16–18]. The most common reason is fracture. Even a minor injury may cause premature closure of the anterior part of the proximal tibial growth plate [16, 17, 19–22]. Possible minor injuries are avulsion of the tibial eminence (Osgood-Schlatter disease), skeletal traction through the proximal tibia, prolonged pressure on the tibial tuberosity by plaster casts, or braces and infectious diseases of the tibia. A few reported occurrences of genu recurvatum deformity in the proximal tibia have been considered idiopathic [19, 20].

Treatment options include open wedge osteotomy of the tibia proximal to the tibial tuberosity [18], closed wedge osteotomy proximal or distal to the tuberosity in association with a fibular osteotomy [23, 24], and open wedge osteotomy in combination with the detachment of the tuberosity [16]. Moroni et al. [16] stressed that in knees in which osteotomy had been carried out proximal to the tuberosity, but in which the patellar tendon had not been detached, the patella was located much too distally. They also stated that when osteotomy was carried out distal to the tuberosity, poor results were obtained because of insufficient correction of the deformity and a prominent anterior curve of the tibial diaphysis was produced. Certain concerns are associated with these acute correcting techniques such as the accuracy of the correction, the patellar height, and the changes in tibial alignment. 

In our case, we performed a high tibial dome osteotomy and tibial tubercle detachment osteotomy. Dome osteotomy offers several advantages. The osteotomy is performed in the broad cancellous metaphyseal region of the tibia. Its large surface area decreases the probability of nonunion as well as it gives the advantage to correct a multiplanar deformity. Because the osteotomy is superior to the tuberosity, the tibial tuberosity can be easily anteriorized in order to correct the extensor mechanism [25]. Dome osteotomy has been used for the treatment of varus deformity in adults [25] due to degenerative knee joint disease as well as in children [26] due to developmental deformity. According to our knowledge, high tibial dome osteotomy has not been used before for the treatment of genu recurvatum. 

Chen et al. [20] state that the ligamentous laxity observed prior to operation resolved as the osteotomy healed. Ligamentous instability should be restored if instability remains after the bone restoration. A staged approach is recommended. Osteotomy first, followed by ligamentous reconstruction (anterior cruciate ligament ACL and posterior cruciate ligament PCL) several months later after the patient has recovered and acceptable correction of the deformity has been identified [27, 28]. Patellar height also must be taken into concern as it is significantly decreased after open wedge osteotomy than closed osteotomy [23].

In recent years, the Ilizarov fixator is widely used due to its advantages of multiplanar stability and versatility to gradually correct deformities in any plane [29, 30]. However the device is bulky, not patient friendly, and social acceptance of the device can be an issue [31, 32]. Many complications have been reported with the Ilizarov fixator, therefore, the surgeon must be experienced with its use. Social adjustments are necessary and the patient must be well disciplined.

As in a conclusion, high level of suspicion is advised to health care professionals, even with a minor knee injury in order to diagnose early this progressive deformity. Posttraumatic genu recurvatum deformity can be well treated with a high tibial dome osteotomy associated with tibial tubercle osteotomy. If ligamentous instability persists, it should be treated separately in a different stage.

## References

[1] Shelton WR, Canale ST (1979). Fractures of the tibia through the proximal tibial epiphyseal cartilage. *Journal of Bone and Joint Surgery A*.

[2] Mann DC, Rajmaira S (1990). Distribution of physeal and nonphyseal fractures in 2,650 long-bone fractures in children aged 0–16 years. *Journal of Pediatric Orthopaedics*.

[3] Peterson CA, Peterson HA (1972). Analysis of the incidence of injuries to the epiphyseal growth plate. *Journal of Trauma*.

[4] Watson-Jones R, Watson-Jones R (1955). Injuries of the knee. *Fractures and Joint Injuries*.

[5] Mudgal CS, Popovitz LE, Kasser JR (2000). Flexon-type Salter-Harris I injury of the proximal tibial epiphysis. *Journal of Orthopaedic Trauma*.

[6] Ogden JA, Tross RB, Murphy MJ (1980). Fractures of the tibial tuberosity in adolescents. *Journal of Bone and Joint Surgery A*.

[7] Manohar Babu KV, Fassier F, Rendon JS, Saran N, Hamdy RC (2012). Correction of proximal tibial recurvatum using the Ilizarov technique. *Journal of Pediatric Orthopaedics*.

[8] Sferopoulos NK (2007). Type v physeal injury. *The Journal of Trauma*.

[9] Ogden JA, Wickland E (1990). Tibia and fibula. *Skeletal Injury in the Child*.

[10] Herring JA (2002). *Tachdjian’s Pediatric Orthopedics*.

[11] Carey J, Spence L, Blickman H, Eustace S (1998). MRI of pediatric growth plate injury: correlation with plain film radiographs and clinical outcome. *Skeletal Radiology*.

[12] Carrino JA, Schweitzer ME (2002). Imaging of sports-related knee injuries. *Radiologic Clinics of North America*.

[13] Futami T, Foster BK, Morris LL, LeQuesne GW (2000). Magnetic resonance imaging of growth plate injuries: the efficacy and indications for surgical procedures. *Archives of Orthopaedic and Trauma Surgery*.

[14] Lohman M, Kivisaari A, Vehmas T, Kallio P, Puntila J, Kivisaari L (2002). MRI in the assessment of growth arrest. *Pediatric Radiology*.

[15] Smith BG, Rand F, Jaramillo D, Shapiro F (1994). Early MR imaging of lower-extremity physeal fracture-separations: a preliminary report. *Journal of Pediatric Orthopaedics*.

[16] Moroni A, Pezzuto V, Pompili M, Zinghi G (1992). Proximal osteotomy of the tibia for the treatment of genu recurvatum in adults. *Journal of Bone and Joint Surgery A*.

[17] Moroni A, Vicenzi G, Ceccarelli F, Binazzi R, Vaccari V (1987). Surgical treatment of genu recurvatum with procurvatum high tibial osteotomy. *Orthopaedic Transactions*.

[18] Lecuire F, Lerat JL, Bousquet G (1980). The treatment of genu recurvatum. *Revue de Chirurgie Orthopedique et Reparatrice de l’Appareil Moteur*.

[19] Segev E, Hendel D, Wientroub S (2002). Genu recurvatum in an adolescent girl: hypothetical etiology and treatment considerations. A case report. *Journal of Pediatric Orthopaedics B*.

[20] Chen LC, Chan YS, Wang CJ (2004). Opening-wedge osteotomy, allografting with dual buttress plate fixation for severe genu recurvatum caused by partial growth arrest of the proximal tibial physis: a case report. *Journal of Orthopaedic Trauma*.

[21] Bellicini C, Khoury JG (2006). Correction of genu recurvatum secondary to Osgood-Schlatter disease: a case report. *The Iowa Orthopaedic Journal*.

[22] Domzalski ME, Lipton GE, Lee D, Guille JT (2006). Fractures of the distal tibial metaphysis in children: patterns of injury and results of treatment. *Journal of Pediatric Orthopaedics*.

[23] Bowen JR, Morley DC, McInerny V, MacEwen GD (1983). Treatment of genu recurvatum by proximal tibial closing-wedge/anterior displacement osteotomy. *Clinical Orthopaedics and Related Research*.

[24] Irwin CE (1942). Genu recurvatum following poliomyelitis: a controlled method of operative correction. *Journal of the American Medical Association*.

[25] Kodkani PS (2007). Dome osteotomy of the proximal tibia for genu varum treated with a new fixation device. *The Journal of Knee Surgery*.

[26] Ogbemudia AO, Bafor A, Ogbemudia PE (2011). Anterior posterior inverted-“U” osteotomy for tibia vara: technique and early results. *Archives of Orthopaedic and Trauma Surgery*.

[27] Noyes FR, Barber-Westin SD, Albright JC (2006). An analysis of the causes of failure in 57 consecutive posterolateral operative procedures. *American Journal of Sports Medicine*.

[28] Noyes FR, Barber SD, Simon R (1993). High tibial osteotomy and ligament reconstruction in varus angulated, anterior cruciate ligament-deficient knees. A two- to seven-year follow-up study. *American Journal of Sports Medicine*.

[29] Causero A, Tcherkes-Zade T, Tcherkes-Zade D, Paschina E (2002). The Ilizarov technique in the treatment of osteoarthritic genu varum. *La Chirurgia degli Organi di Movimento*.

[30] Niedzielski K, Fabiś J, Synder M (1999). Application of the Ilizarov apparatus in the treatment of knee osteoarthritis. *Chirurgia Narzadów Ruchu i Ortopedia Polska*.

[31] Catagni MA, Guerreschi F, Ahmad TS, Cattaneo R (1994). Treatment of genu varum in medial compartment osteoarthritis of the knee using the Ilizarov method. *Orthopedic Clinics of North America*.

[32] Velazquez RJ, Bell DF, Armstrong PF, Babyn P, Tibshirani R (1993). Complications of use of the Ilizarov technique in the correction of limb deformities in children. *Journal of Bone and Joint Surgery A*.

